# Structure and Crystallization of High-Calcium, CMAS Glass Ceramics Synthesized with a High Content of Slag

**DOI:** 10.3390/ma15020657

**Published:** 2022-01-16

**Authors:** Lishun Chen, Yuting Long, Mingkai Zhou, Huaide Wang

**Affiliations:** State Key Laboratory of Silicate Materials for Architectures, Wuhan University of Technology, 122 Luoshi Road, Wuhan 430070, China; chenlishun@whut.edu.cn (L.C.); ytlong@whut.edu.cn (Y.L.); whd@whut.edu.cn (H.W.)

**Keywords:** high-calcium CMAS, glass ceramics, ferromanganese slag, structure, crystallization

## Abstract

In this work, more than 70 wt % of ferromanganese slag (containing 40 wt % CaO) was used to synthesize high-calcium, CaO-MgO-Al_2_O_3_-SiO_2_ (CMAS) glass ceramics. The effect of SiO_2_/CaO on the structure, crystallization behavior and microstructure of high-calcium, CMAS, slag glass ceramics was studied by IR, NMR, DSC, XRD and SEM. The results showed that in the high-calcium, CMAS glass ceramics, the main existing forms of silicon–oxygen tetrahedra (Q^n^) were Q^0^ and Q^1^. With the increase in the SiO_2_/CaO, Q^n^ changed from Q^0^ and Q^1^ (main units) to Q^1^ (main units) and Q^2^, and then to Q^1^ and Q^2^ (main units). The polymerization degree of Q^n^ changed from low to high, making the glass more stable, which led to the increase in crystallization temperature and the decrease in crystallization kinetic constant (k) and frequency factor (υ). At the same time, the change in the Q^n^ structure resulted in a gradual change to the main crystal, from akermanite to diopside–wollastonite.

## 1. Introduction

Ferromanganese is an important mineral resource and has a significant influence on the construction industry. The smelting efficiency of ferromanganese is significantly affected by the smelting process and its ore grade. In the smelting process, in order to improve the smelting efficiency, the ratio of CaO/SiO_2_ needs to be controlled and kept in the range 1.3~1.5 [1,2,3]. Therefore, ferromanganese slag is a slag with high CaO content. In China, ferromanganese slag has a higher content of CaO as the grade of manganese ore is lower than that of the average for the rest of the world [4,5]. According to statistics, the annual emissions of ferromanganese slag in China were about 18 million tons in 2017, the majority of the ferromanganese slag ended up in landfill [6,7]. The minority of the ferromanganese slag was used in the building-materials industry, and its dosage did not exceed 30 wt % [8,9]. Hence, high-volume utilization of ferromanganese slag is an important factor affecting the healthy and orderly development of the ferroalloy industry, which also leads to significant economic and environmental benefits.

Slag glass ceramics are environmentally friendly materials prepared using slag as the main raw material, which is a low-cost and high-value-added material. CaO–MgO–Al_2_O_3_–SiO_2_ (CMAS) is one of the important components in the field of glass–ceramic materials, whose main precipitation is diopside. Due to the crystal structure of diopside, CMAS-system glass ceramics show advantages, which include high strength and excellent chemical resistance [10,11,12]. CMAS systems are often used to design slag glass ceramics, because its main chemical composition is close to that of slag [13,14,15].

In the field of glass ceramics, using ferromanganese slag to prepare architectural glass ceramics is rarely studied. This is because, compared with other slags, the ferromanganese slag cannot be used at such a high content in traditional CMAS systems [16,17,18]. In traditional CMAS glass ceramics, the CaO content is generally not higher than 20 wt % for the sake of diopside crystallization [19,20]. This means that the utilization rate of high-calcium slag, such as ferromanganese slag (CaO ≥ 40 wt %), cannot exceed 50 wt%. When using ferromanganese slag at high dosages, the CaO content in the glass composition will be higher than that of traditional CMAS systems. Therefore, studying the glass structure and crystallization behavior of high-calcium CMAS systems is conducive to realizing the use of higher contents of high calcium slag.

SiO_2_ and CaO are important components of glass, and the ratio of SiO_2_/CaO will have a significant impact on the basic structure and crystallization behavior of the glass. Li et al. found that an increase in calcium oxide content will increase the content of low-polymerization silica tetrahedra in glass structures [21]. Jia et al. found that when the content of CaO is less than 32 wt %, adjusting the ratio of SiO_2_/CaO from 1.34 to 2.44 caused a series of changes in the viscosity, crystal form, structure and mechanical properties of the glass [22]. Wang et al. prepared glass ceramics by using 37~56 wt % blast furnace slag, and when the SiO_2_/CaO ratio was reduced from 3.33 to 1.58, the crystal changed from diopside to akermanite [23]. Hence, it is of critical importance to study the influence of the SiO_2_/CaO ratio in high-calcium CMAS systems.

In this work, ferromanganese slag was used as the main raw material to prepare high-calcium CMAS glass ceramics, and the slag content was higher than 70 wt %. Infrared spectroscopy (IR), nuclear magnetic resonance (NMR), differential scanning calorimeter (DSC), X-ray diffraction (XRD) and field-emission scanning electron microscopes (SEM) have been used to study the effects of the SiO_2_/CaO ratio on the structure and crystallization of high-calcium CMAS glass ceramics.

## 2. Materials and Methods

### 2.1. Materials Preparation

The ferromanganese slag and bauxite used in the experiment were obtained from YW Ferroalloy Factory in Lvliang, Shanxi Province, China. The oxide compositions of ferromanganese slag and bauxite are listed in Table 1.

### 2.2. Glass Composition

Ferromanganese slag was used as the main raw material and bauxite as the auxiliary material. By adding SiO_2_, Al_2_O_3_, MgO and other chemical reagents, the mass ratio of Al_2_O_3_, MgO, Fe_2_O_3_ and MnO were kept unchanged. Each sample was premilled by ball milling and kept at 1500 °C for 2 h. The samples were quenched with water, ground by a vibrating mill and passed through a 200 mesh sieve for subsequent testing. Specific oxide composition is shown in Table 2.

### 2.3. Characterizations

Infrared spectra of the sample were detected by infrared spectrometer (Nexus, Therno Nicolet, USA) in the range 400–2000 cm^−1^.

^29^Si magic angle spinning nuclear magnetic resonance (MAS-NMR) spectra were obtained at 79.49 MHz using a 400 MHz AVANCE III spectrometer (Bruker, Switzerland, 9.8 T, wide-bore, using 7 mm zirconia rotors spinning at 5 kHz at ambient temperature). The ^29^Si MAS-NMR samples were spun with a recycle delay time of 5 s and the number of scans was 1024. Chemical displacement was quoted in parts per million (ppm) from tetramethylsilane (TTMS). 

The glass crystallization temperature was collected by a differential scanning calorimeter (DSC, Netzsch STA 449F3, Germany) with alumina as the reference. The temperature was scanned over a range from room temperature to 1000 °C at a heating rate of 5, 10, 15 and 20 °C/min.

Crystalline phases of glass ceramics were identified by X-ray diffraction (XRD, D/MAX-RB Rigaku, Japan) with Cu radiation (λ = 0.15405 μm) at 40 kV and 40 mA. The diffraction patterns were recorded from 10° to 70°, with a scanning speed of 2°/min.

The microstructure of the samples was observed on fresh fracture surfaces using a field emission scanning electron microscope (SEM, JSM-IT300, JEOL, Japan). The fracture surface was chemically etched by 4% HF for 40 s. The internal structural properties were determined by field-emission, high-resolution transmission electron microscope (TEM, JEM-2100F, JEOL, Japan). 

## 3. Results

### 3.1. Characterization of Basic Glass

**Figure 1:**
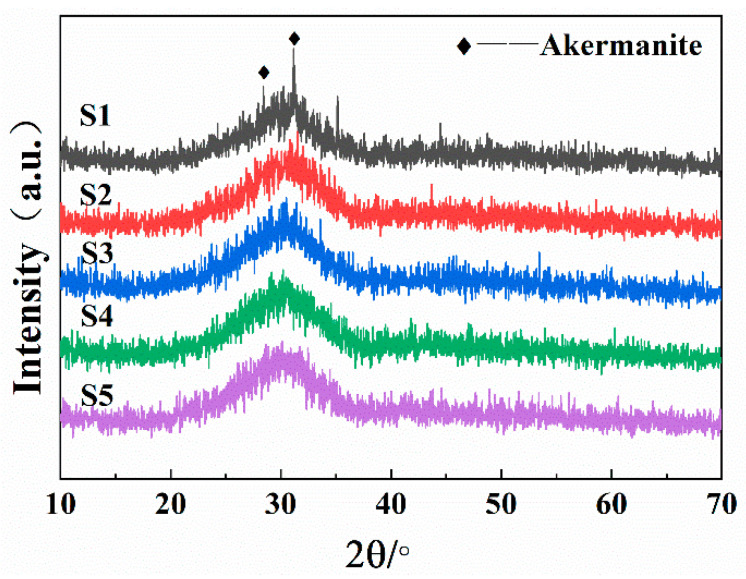
XRD patterns of quenched samples with different SiO_2_/CaO ratios.

Figure 1 shows the XRD patterns of quenched samples with different SiO_2_/CaO ratios. Obvious crystallization peaks can be seen in S1, which contained 100% slag. This indicated that the raw-slag melt was prone to crystallize. This is because SiO_2_, as a glass network former, mainly participates in the composition of the glass network structure. The stable silicon–oxygen tetrahedral structure will increase the difficulty of particle migration, thereby reducing the tendency of crystallization [24]. CaO, as a network modifying oxide, can provide free oxygen (O_nb_) to destroy the glass network structure, thereby reducing the degree of polymerization of the glass network structure [25,26]. The decrease in polymerization degree was conducive to the migration of particles, thereby increasing the tendency of crystallization. The SiO_2_/CaO ratio of S1 was 0.98. this low silicon-to-calcium ratio made the glass network structure unstable, so S1 had a greater tendency to crystallize. As the ratio of SiO_2_/CaO increased, there was no obvious crystallization peak in S2–S5. This showed that the increase in the SiO_2_/CaO ratio was conducive to stabilizing the glass network structure.

**Figure 2:**
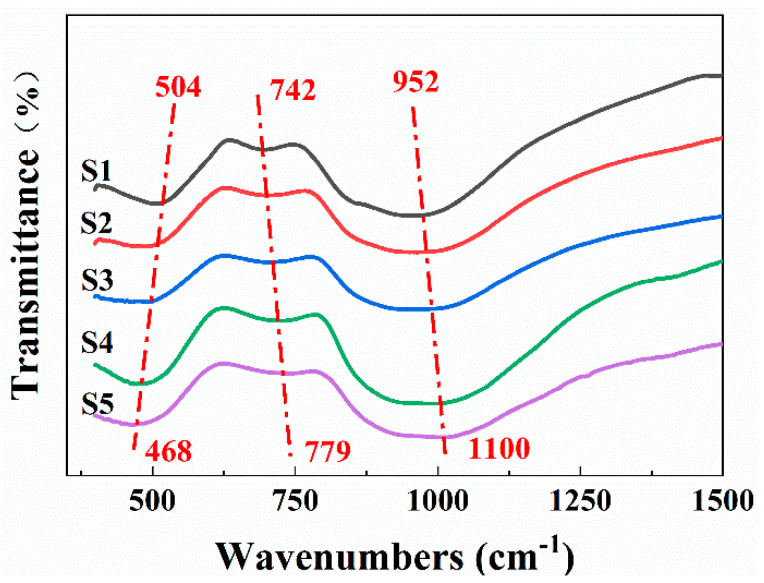
Infrared spectra of S1–S5.

Infrared spectroscopy (IR) can reflect the transition of vibrational and rotational energy levels in specific molecules by detecting the absorption of infrared light. Figure 2 shows the infrared spectra of S1–S5. The common structures in glass are [AlO_4_], [AlO_6_] and [SiO_4_], and the tetrahedral structure can be connected by bridge oxygen (O_b_) to form anionic groups with a higher degree of polymerization. Corresponding characteristic vibrations of CAMS glass are listed in Table 3 [27,28].

It can be seen from Figure 2 that there are three obvious absorption peaks located around 450~550 cm^−1^, 640~800 cm^−1^ and 850~1150 cm^−1^, and the peak positions shifted significantly. As shown in Table 3, the peak around 450~550 cm^−1^ was ascribed to the Si-O_b_-Si bending vibration and Al-O bending vibration in [AlO_6_]. The peak shifts to lower wavenumbers indicated that the bending vibration of Si-O_b_-Si was strengthened. Therefore, the structure of Si-O_b_-Si and the amount of O_b_ in the glass increased. The peak around 640~800 cm^−1^ was ascribed to the Al-O bending vibration in [AlO_4_], Si-O_b_-Si symmetric stretching vibration and O-Si-O symmetric bending vibration. The peak around 850~1150 cm^−1^ could be assigned to the Si-O_nb_ anti-symmetric stretching vibration and Si-O_b_-Si anti-symmetric stretching vibration. Both peaks tended to shift to higher wavenumbers, which also explained the increase in the amount of O_b_. The IR results indicated that the increase in the SiO_2_/CaO ratio could effectively form O_b_.

### 3.2. The Influence of SiO_2_/CaO on the Silicon–Oxygen Tetrahedron Structure

In silicon-based glass, the basic structural units are composed of a spin silicon–oxygen tetrahedron structure, in which a silicon atom is connected to four oxygen atoms. The silicon–oxygen tetrahedral structure with a silicon atom at the center can be denoted as Q^n^, where Q represents the silicon atom and n represents the number of O_b_ connected to the silicon atom. IR results showed that the number of O_b_ increased, and the polymerization degree of silicon–oxygen tetrahedra increased. However, a specific existing forms of the Q^n^ structures in the high-calcium CMAS systems was still unclear. As NMR is a technique commonly used for short-range and medium-range structure analysis of glass, including ^29^Si, ^27^Al, ^17^O, the study was further carried out by NMR. The relative contents of each silicon–oxygen tetrahedral structure could be obtained by fitting and analyzing the measured curve [29,30].

**Figure 3:**
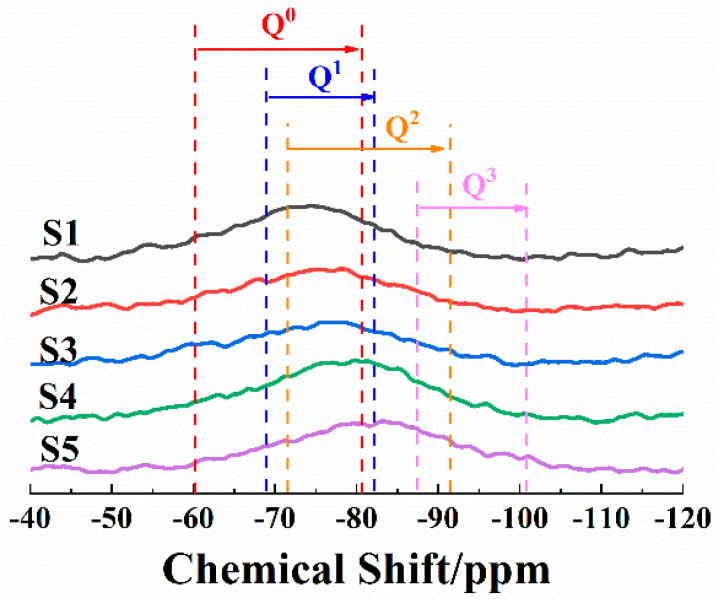
^29^Si NMR spectra of S1–S5.

It can be seen from Figure 3 that the center of the ^29^Si NMR peak was around −70~−80 ppm, indicating that the silicon–oxygen tetrahedra structures in the high-calcium CMAS systems were mainly Q^0^, Q^1^ and Q^2^ [31,32,33]. This was different from traditional low-calcium CMAS systems, whose main existing forms of silicon–oxygen tetrahedra are Q^1^, Q^2^ and Q^3^ [34,35]. With the increase in the SiO_2_/CaO ratio, the chemical shift of the S1–S5 peak centers became smaller. This indicated that the existing forms of the silicon–oxygen tetrahedra gradually changed from low polymerization degrees to high polymerization degrees, which was consistent with IR results.

**Figure 4:**
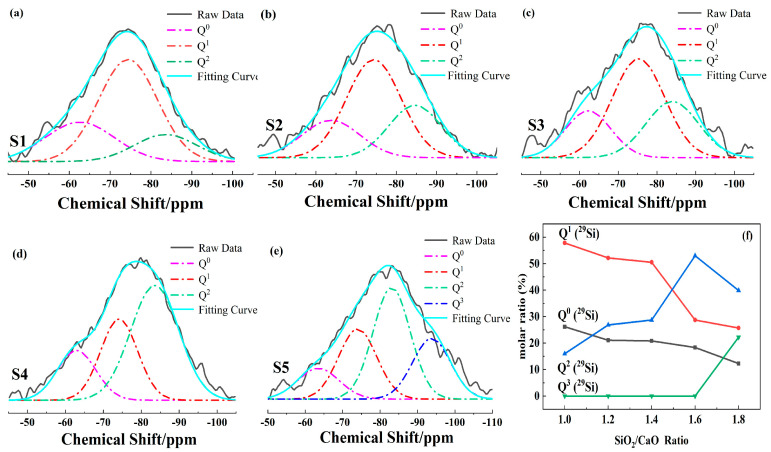
(**a**–**e**) Typical deconvolution of ^29^Si NMR spectra for S1–S5. (**f**) Relative peak-area ratio of different Qn changes with the SiO_2_/CaO ratio.

Fourier transformation was performed to smooth the curves. The spectra intensities were normalized, and the peaks were deconvoluted (the peak centers were at −64 ppm for Q^0^, −74 ppm for Q^1^, −83 ppm for Q^2^, −94 ppm for Q^3^). Figure 4a shows a typical deconvolution for S1–S5. Figure 4b shows the relative peak-area ratio of different Q^n^ changing with the SiO_2_/CaO ratio.

As shown in Figure 4, the main forms of silicon–oxygen tetrahedra in S1 were Q^0^ and Q^1^ (main units). This was because the SiO_2_/CaO ratio of S1 was 0.98, and the molar ratio of oxygen atoms to silicon atoms was larger than 3.5, which made the silicon–oxygen tetrahedron unable to form a high degree of polymerization anion group [36]. As the ratio of SiO_2_/CaO increased (Figure 4f), the content of Q^0^ and Q^1^ gradually decreased, and the content of Q^2^ gradually increased. This was because, with the increase in SiO_2_/CaO, the free oxygen introduced by CaO decreased, whereas the Si atoms that could form silicon–oxygen tetrahedra increased. Low-polymerization-degree [SiO_4_] needs to be bonded with oxygen atoms to form glass structures with a high polymerization degree, so as to achieve a balance of valence. When SiO_2_/CaO changed from 1.6 to 1.8, the content of Q^2^ was reduced, because Q^3^ (higher degree of polymerization) was formed in the glass.

In high-calcium CMAS systems, an increase in the SiO_2_/CaO ratio will cause three significant changes in the main existing form of Q^n^. When SiO_2_/CaO increased from 0.98 to 1.2, the main forms of silicon–oxygen tetrahedra changed from Q^0^ and Q^1^ (main units) to Q^1^ (main units) and Q^2^. When the SiO_2_/CaO ratio increased from 1.4 to 1.6, the main forms of silicon–oxygen tetrahedra changed from Q^1^ (main units) and Q^2^ to Q^1^ and Q^2^ (main units). When the SiO_2_/CaO ratio reached 1.8, a glass structure with a higher degree of polymerization, i.e., Q^3^, began to appear.

### 3.3. The Effect of SiO_2_/CaO on Crystallization Behavior

**Figure 5:**
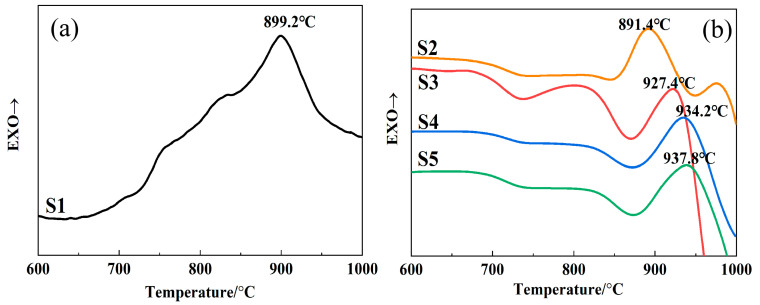
DSC curves of (**a**) S1 and (**b**) S2–S5 at heating rate of 10 °C/min.

Figure 5 shows the DSC curves of the samples with different SiO_2_/CaO ratios, at a heating rate of 10 °C/min. The DSC curves of the original slag sample and the modified samples are depicted respectively. As for S1, the DSC curve exhibited a low initial exothermic temperature and a broad and coarse exothermic peak in the range 700~1000 °C, which indicated that S1 had a wide crystallization range. According to XRD pattern of the water-quenched samples (Figure 1), S1 had already undergone crystallization during the cooling process. In the subsequent crystallization process, when the temperature rose above the glass transition temperature, these precipitated crystals would grow further. Therefore, the initial temperature of the exothermic process, caused by crystal growth, was lower. At the same time, a new nucleation process and crystal growth process would occur, so the entire crystallization process lasted for a long period of time, which caused a wide exothermic peak on the DSC curve.

Compared to S1, the exothermic peak width of S2–S5 were significantly narrowed and the initial exothermic temperature was increased from 700 °C to 850 °C. This was because the rapid crystallization of the slag was solved as the SiO_2_/CaO ratio increased. In detail, S2–S5 needed to undergo a nucleation process before the crystallization process during the heating process. Therefore, the initial exothermic temperature was significantly increased.

The narrower crystallization peak indicated that the crystallization process of the samples occurred in a narrow temperature range, which was conducive to controlling the appropriate crystallization temperature so that the uniform crystal grain could be precipitated. For S2–S5, with the increase in the SiO_2_/CaO ratio, the shape of the crystallization peak did not change significantly, but the temperature of the crystallization peak kept shifting to higher temperatures. This was because the silicon–oxygen tetrahedron changed from Q^1^ (main units) to Q^2^ (main units) as the ratio of SiO_2_/CaO increased. Q^n^ changed a from low polymerization degree to a higher polymerization degree. The stable glass structure made it more difficult for particles to migrate during the crystallization process, so the corresponding crystallization peak temperature increased.

In order to investigate the effect of the SiO_2_/CaO ratio on the crystallization mechanism of glass, the crystallization activation energy (*E_c_*) of S1–S5 was evaluated by non-isothermal DSC curves. The crystallization peak temperatures (*T_p_*) of S1–S5 are shown in Table 4. The non-isothermal crystallization kinetics of glass can be described by the Kissinger equation [37].

$$\ln\left(\frac{T_{p}^{2}}{\alpha }\right)=\frac{E_{c}}{RT_{p}}+\ln\left(\frac{E_{c}}{R}\right)-\ln v\;$$

**Figure 6:**
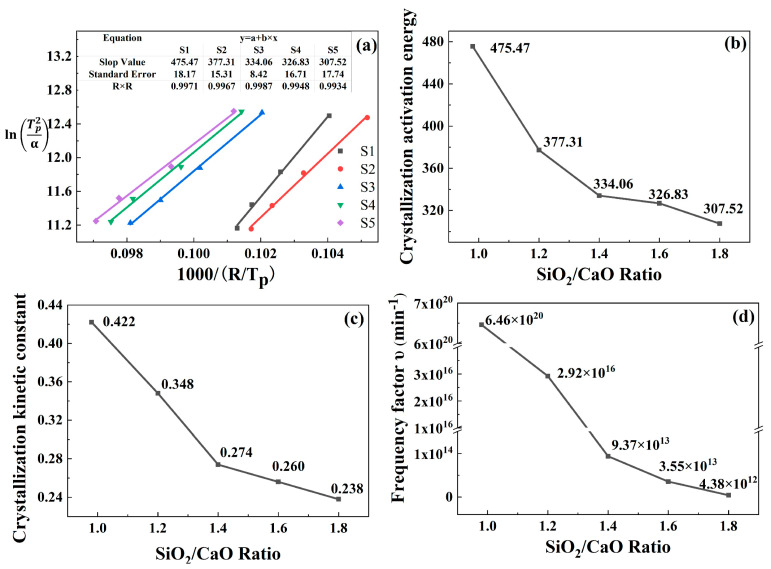
(**a**) Relationship between $\ln\;\left(T_{p}{}^{2}/\alpha \right)$ and $1000/\left(R\times T_{p}\right)$, relationship between SiO_2_/CaO ratio with *E_c_* (**b**), *k* (**c**), *υ* (**d**).

The crystallization kinetic constant (*k*) is related to the crystallization peak temperature and can be described by the Arrhenius equation.

$$k=\upsilon \times \exp\left(-\frac{E_{c}}{RT_{p}}\right)$$

where *T_p_* is the crystallization peak temperature, *α* is the heating rate and *R* is the gas constant. Figure 6a shows the correlation between$\;\ln\;\left(T_{p}{}^{2}/\alpha \right)$ and $1000/\left(R\times T_{p}\right)$ of S1–S5. The *E_c_*, *k* and *υ* were calculated, and the relationship of *E_c_*, *k* and *υ* with SiO_2_/CaO was plotted, as shown in Figure 6b–d.

It was found that as the ratio of SiO_2_/CaO increased, the activation energy of the sample gradually decreased. Similar results were found by Deng when a high content of stainless-steel slag (more than 50 wt %) was used [38]. Cheng et al. pointed out that activation energy could not be directly used to judge the stability of glass. It was necessary to introduce the crystallization kinetic constant k and frequency factor *υ* to evaluate the thermal stability of glass [39]. Hu et al. also pointed out that *k* and *υ* could reflect the thermal stability and internal structure of the glass [40]. The stability of the glass increased as the values of *k* and *v* decreased. As the ratio of SiO_2_/CaO increased, the kinetic constant and frequency factor of the sample decreased, indicating that the glass structure became more stable, which was consistent with the IR and NMR results.

**Figure 7:**
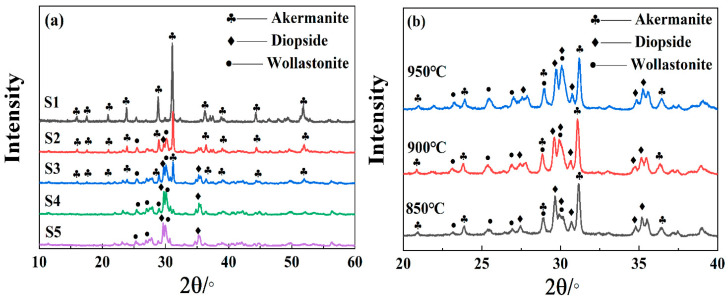
(**a**) XRD patterns of S1–S5 crystallized at 950 °C for 1 h. (**b**) XRD patterns of the S3 heated at different temperatures for 1 h.

Figure 7a,b are the XRD patterns of S1–S5 crystallized at 950 °C for 1 h and S3 heated at 850 °C, 900 °C and 950 °C for 1 h, respectively. As shown in Figure 7a, only akermanite (Ca_2_MgSi_2_O_7_) was precipitated in S1. With the increase in the SiO_2_/CaO ratio, the content of akermanite gradually decreased until it disappeared. The main crystal phase of the sample gradually changed from akermanite to diopside–wollastonite (CaMgSi_2_O_6_-CaSiO_3_), and the amount of diopside gradually increased with the increase in SiO_2_/CaO ratio.

By comparing crystal form and the content of Q^n^, it was found that the content of akermanite changed with Q^1^ and the content of diopside–wollastonite changed with Q^2^. When SiO_2_/CaO increased from 0.98 to 1.2, the main forms of the silicon–oxygen tetrahedra changed from Q^0^ and Q^1^ (main units) to Q^1^ (main units) and Q^2^. The appearance of Q^2^ corresponded to the precipitation of diopside–wollastonite. When SiO_2_/CaO increased from 1.4 to 1.6, the main forms of the silicon–oxygen tetrahedra changed from Q^1^ (main units) and Q^2^ to Q^1^ and Q^2^ (main units), which corresponded to the disappearance of akermanite. This was because the transformation of Q^n^ affected the precipitation of crystals [41]. 

**Figure 8:**
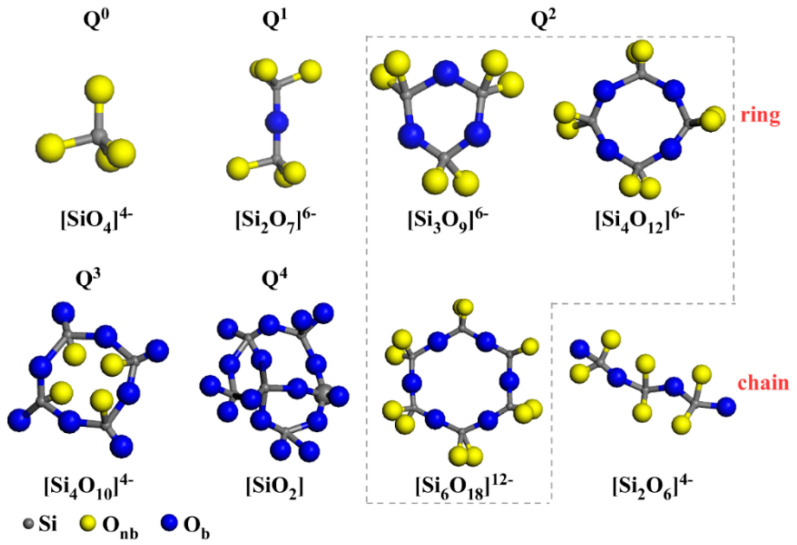
The correlation between Qn and basic silicate structural units.

As shown in Figure 8, Q^n^ corresponded to different anion group structures. Q^1^ contained a bridging oxygen, which corresponded to the anionic group structure of [Si_2_O_7_]^6−^. When the SiO_2_/CaO ratio was low, the main existing form of the silicon–oxygen tetrahedron in the glass structure was Q^1^, which made it easier to precipitate crystals with the anionic group structure of [Si_2_O_7_]^6−^, that is, akermanite. Q^2^ contained two bridge oxygens, which could be formed in different anionic group structures such as [Si_3_O_9_]^6−^, [Si_4_O_12_]^8−^, [Si_6_O_18_]^12−^ and [Si_2_O_6_]^4−^. When the main form of silicon–oxygen tetrahedron in the glass structure was Q^2^, it was easier to precipitate crystals (wollastonite and diopside) with the corresponding anion group structure. Therefore, when the tetrahedral structure in the glass changed, the crystal form corresponding to different SiO_2_/CaO changed at the same time.

It can be seen from Figure 7b that the diopside phase was first precipitated in the sample during the crystallization process, and the wollastonite phase was gradually precipitated as the temperature rose. This was because Q^n^ underwent structural transformation during the heating process, such as Q^n^↔Q^n^^−1^ + Q^n+1^ [42]. The anion group of diopside (CaMgSi_2_O_6_) and wollastonite (CaSiO_3_) both belong to Q^2^. As for [Si_3_O_9_]^6−^, [Si_4_O_12_]^8−^ and [Si_6_O_18_]^12−^, the formation of these ring-like structures was difficult, and required breaking, bonding and bending of several Si–O bonds from Q^1^. However, [Si_2_O_6_]^4−^ is a chain structure, so its transformation from Q^1^ required the breaking and bonding of fewer bonds. Therefore, diopside was easier to crystallize than wollastonite.

**Figure 9:**
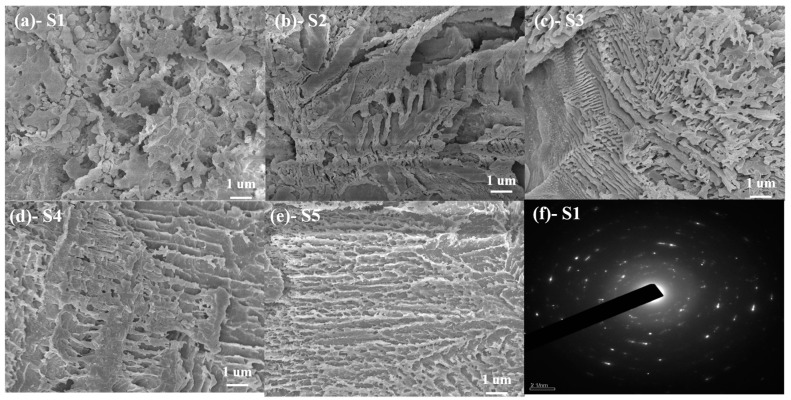
(**a**–**e**) SEM images of S1–S5 treated at 950 °C for 1 h. (**f**) TEM image of S1 quenched by water.

Figure 9a–e shows the microstructure of S1–S5, which were made from powders kept at 950 °C for 1 h. Figure 9f shows the TEM image of S1. As shown in Figure 9a, herringbone crystals, with a grain size of approximately 1 μm, and sphere particles, with grain size of 0.2 μm, could be found in S1. These crystals with different grain sizes were both akermanite, according to the XRD result. This indicated that the growth process of grains in S1 differed. The TEM image of S1 showed that there were obvious diffraction spots in the water-quenched sample, indicating that crystals had already precipitated during the cooling process at the low SiO_2_/CaO ratio. From DSC, the growth of existing crystals could occur at a low temperature. However, the new crystal needed to form a nucleus first, and then grow, so there was a significant difference in crystal sizes. For S2, the main crystal phase was still akermanite, so herringbone crystals could be seen in SEM image. The increase in the SiO_2_/CaO ratio stabilized the glass structure, so the grain size in S2 was more uniform than that in S1. The main crystal form of S3 was akermanite and diopside–wollastonite, so a variety of crystals could be clearly seen in the SEM image of S3. When the SiO_2_/CaO ratio was further increased, there was almost no akermanite in the sample, so the SEM mainly existed in the form of dendrites.

## 4. Conclusions

(1) In the high-calcium CMAS systems, the type of crystals precipitated in the sample was closely related to Q^n^. When the ratio of SiO_2_/CaO was 0.98, the main forms of Q^n^ were Q^0^ and Q^1^ (main units). At this time, only akermanite was precipitated. As the ratio of SiO_2_/CaO increased, the main forms of Q^n^ changed from Q^0^ and Q^1^ (main units) to Q^1^ (main units) and Q^2^. The sample began to precipitate diopside–wollastonite. When the SiO_2_/CaO ratio was greater than 1.6, the main existing form of the silicon-oxygen tetrahedron changed from Q^1^ (main units) and Q^2^ to Q^1^ and Q^2^ (main units). At this time, akermanite in the sample disappeared, and only diopside–wollastonite was precipitated.
(2) A low SiO_2_/CaO ratio reduced the stability of the glass, and caused rapid crystallization during the melt–cooling process in high-calcium CMAS systems. As the ratio of SiO_2_/CaO increased, the anionic groups in the glass changed from a low polymerization degree to a high polymerization degree. The glass stability was obviously improved. The crystallization peak temperature of the sample continuously increased, and the crystallization kinetic constant k and the frequency factor υ gradually decreased.
(3) In high-calcium CMAS systems, the rapid crystallization problem in the cooling process, caused by low SiO_2_/CaO, caused significant differences in grain size. As the ratio of SiO_2_/CaO increased, the homogeneity of the crystal improved, and the crystal changed from herringbone shape (akermanite) to dendritic shape (diopside–wollastonite).

## Figures and Tables

**Table 1:** Oxide composition of main materials (wt %).

Material	LOSS	SiO_2_	Al_2_O_3_	CaO	MgO	Fe_2_O_3_	MnO
Ferromanganese slag	0.27	40.87	4.46	41.78	6.87	0.26	3.99
Bauxite	12.83	34.84	43.52	0.76	0.29	2.61	0.04

**Table 2:** Oxide composition of glass ceramics.

Sample	SiO_2_/CaO	Al_2_O_3_	SiO_2_	CaO	MgO	Fe_2_O_3_	MnO
S1	0.98	4.5	40.9	41.8	6.9	0.26	4.0
S2	1.20	4.5	45.11	37.59	6.9	0.26	4.0
S3	1.40	4.5	48.24	34.46	6.9	0.26	4.0
S4	1.60	4.5	50.89	31.81	6.9	0.26	4.0
S5	1.80	4.5	53.16	29.53	6.9	0.26	4.0

**Table 3:** Characteristic vibrations of CAMS glass.

Wavenumber/cm^−1^	Corresponding Characteristic Vibration
430~550	Bending vibration of Si-O_b_-Si
520	The Al-O bending vibration in [AlO_6_]
700	The Al-O bending vibration in [AlO_4_]
760	Symmetric stretching vibration of Si-O_b_-Si
780~800	Symmetric bending vibration of O-Si-O
950	Anti-symmetric stretching vibration of Si-O_nb_
1020~1060	Anti-symmetric stretching vibration of Si-O_b_-Si

**Table 4:** $T_{p}\;$of S1–S5 at different heating rates.

Sample	$T_{p}/{}^{\circ}C\cdot \min^{-1}$			
	5 °C·min^−1^	10 °C·min^−1^	15 °C·min^−1^	20 °C·min^−1^
S1	882.8	899.2	909.1	914.2
S2	870.3	891.4	902.1	909.3
S3	905.6	927.4	941.7	952.8
S4	912.7	934.2	951.9	960.1
S5	915.4	937.8	957.1	965.8
